# Atypical Burkholderia Cepacia Resistance to Ceftazidime/Avibactam and Co-trimoxazole: A Case of Open Wound Contamination and Persistent Bacteremia

**DOI:** 10.7759/cureus.15836

**Published:** 2021-06-22

**Authors:** Fidelis Uwumiro, Ehizogie Edigin, Victory Okpujie

**Affiliations:** 1 Internal Medicine, Our Lady of Apostles Hospital, Akwanga, NGA; 2 Internal Medicine, John H. Stroger, Jr. Hospital of Cook County, Chicago, USA; 3 Internal Medicine, Central Hospital, Benin City, NGA

**Keywords:** ceftazidime/avibactam resistance, meropenem, burkholderia cepacia complex, peripheral contamination, actinomycetes co-infection, autogenous skin grafting

## Abstract

Wound contamination and subsequent colonization by microbes can significantly impair tissue repair and lead to the development of chronic non-healing ulcers. Atypical *Burkholderia*and *Actinomycetes*bacterial species are common in cases of soil contamination of open wounds leading to a complex infection that is both difficult to diagnose and treat. Despite much research on the involvement of atypical organisms, including *Burkholderia*and *Actinomycetes*, in antibiotic resistance, there is no consensus on the timeline from contamination to infection and on an algorithm for early diagnosis and management. Thus, the ways in which these organisms interact in settings of co-infection and contribute to cross-resistance remains unclear. The generally low index of clinical suspicion for atypical microbial infections and the absence of clear diagnostic protocols have multiple consequences, ranging from excessive reliance on pathology, delayed treatment, expensive and ineffective investigations and treatment, and progressive wound sepsis and morbidity.

We are reporting a case of *Burkholderia cepacia *infection, co-infection with *Actinomyces* spp., and resistance to ceftazidime/avibactam and co-trimoxazole in a 28-year-old previously healthy farmer following soil contamination of an open wound. This is one of only a few reported cases of *Burkholderia *resistance to ceftazidime/avibactam and the first reported case of*B.cepacia *bacteremia due to peripheral contamination.

## Introduction

The Burkholderia cepacia complex refers to a group of gram-negative bacteria known to contribute to poor clinical outcomes in cystic fibrosis patients [1]. B. cepacia is associated with a wide range of infections, including urinary tract infections, respiratory tract infections, and bacteremia. These infections can be difficult to treat owing to high intrinsic resistance. The genus Burkholderia generally shows susceptibility to co-trimoxazole, ceftazidime, cefepime, and carbapenems [2].

## Case presentation

A male 28-year-old previously-healthy farmer was admitted from the outpatient department with a five-day history of fever, malaise, an intermittent cough, mild shortness of breath, left leg pain, and a four-week history of a post-traumatic leg ulcer sustained while working on his farm. His past medical history included an appendectomy four years earlier. Since the onset of the ulcer, he had received treatment with oral amoxicillin/clavulanate and later metronidazole. At the time of admission for in-patient care, his body temperature was 38.2oC, his pulse was 112 bpm (beats per minute) and his blood pressure was 90/60 mmHg. Empiric treatment with intravenous ceftazidime/avibactam, metronidazole, and clindamycin was commenced. A physical examination revealed a non-healing malodorous ulcer with unhealthy granulation tissue and copious slough. Tests revealed an elevated white cell count, anemia, and mild elevation of transaminases. A chest X-ray showed bilateral patchy opacities. However, X-rays of the patient’s left leg were unremarkable. He was admitted with a diagnosis of septic shock linked to the open wound and managed with the aforementioned antibiotics, as well as intravenous fluids, packed red cells, analgesics, and regular diet as tolerated. Blood samples and wound swabs were sent for culture and sensitivity testing.

On the fifth day of admission, the patient’s blood pressure, cough, and breathlessness had improved, but he was still febrile, mildly dyspneic, and lethargic. The results of his blood and wound swab cultures were not yet available.

**Figure 1:**
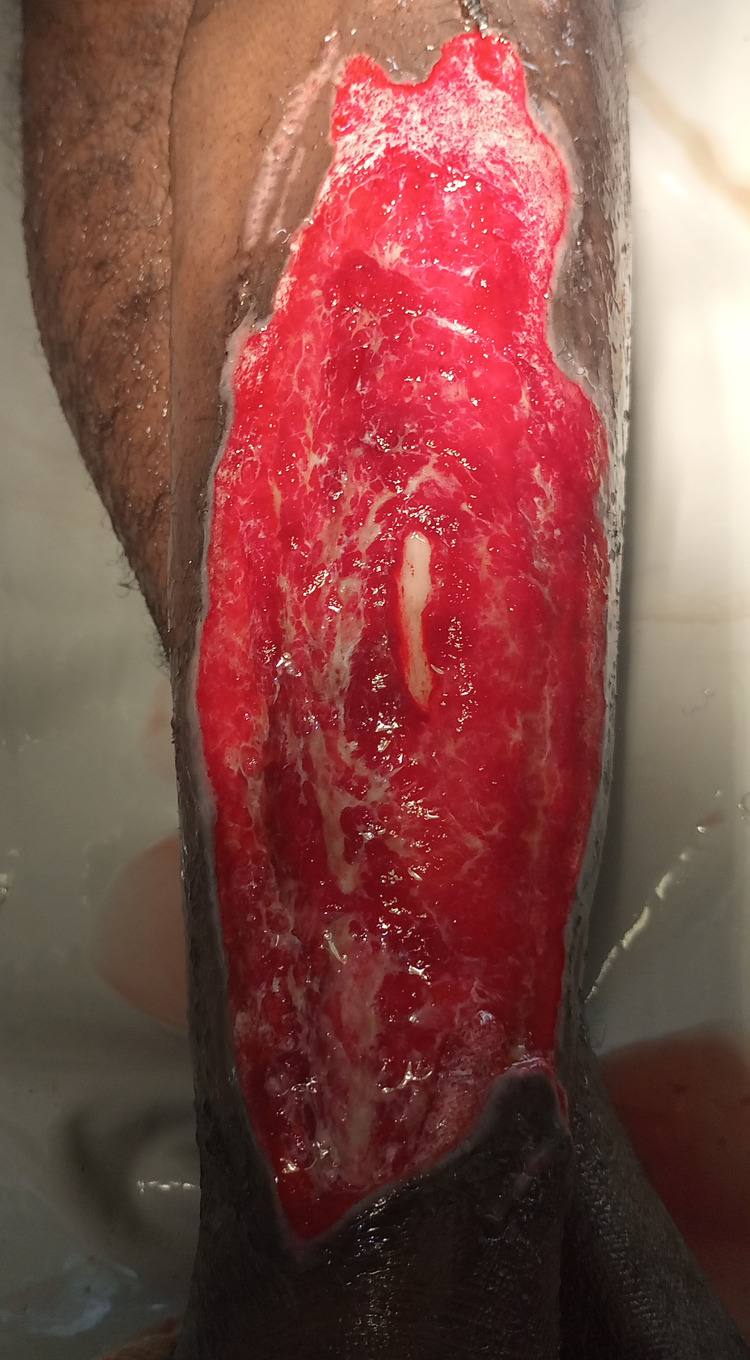
Extensive tissue invasion seen after irrigation and debridement of leg ulcer

**Figure 2:**
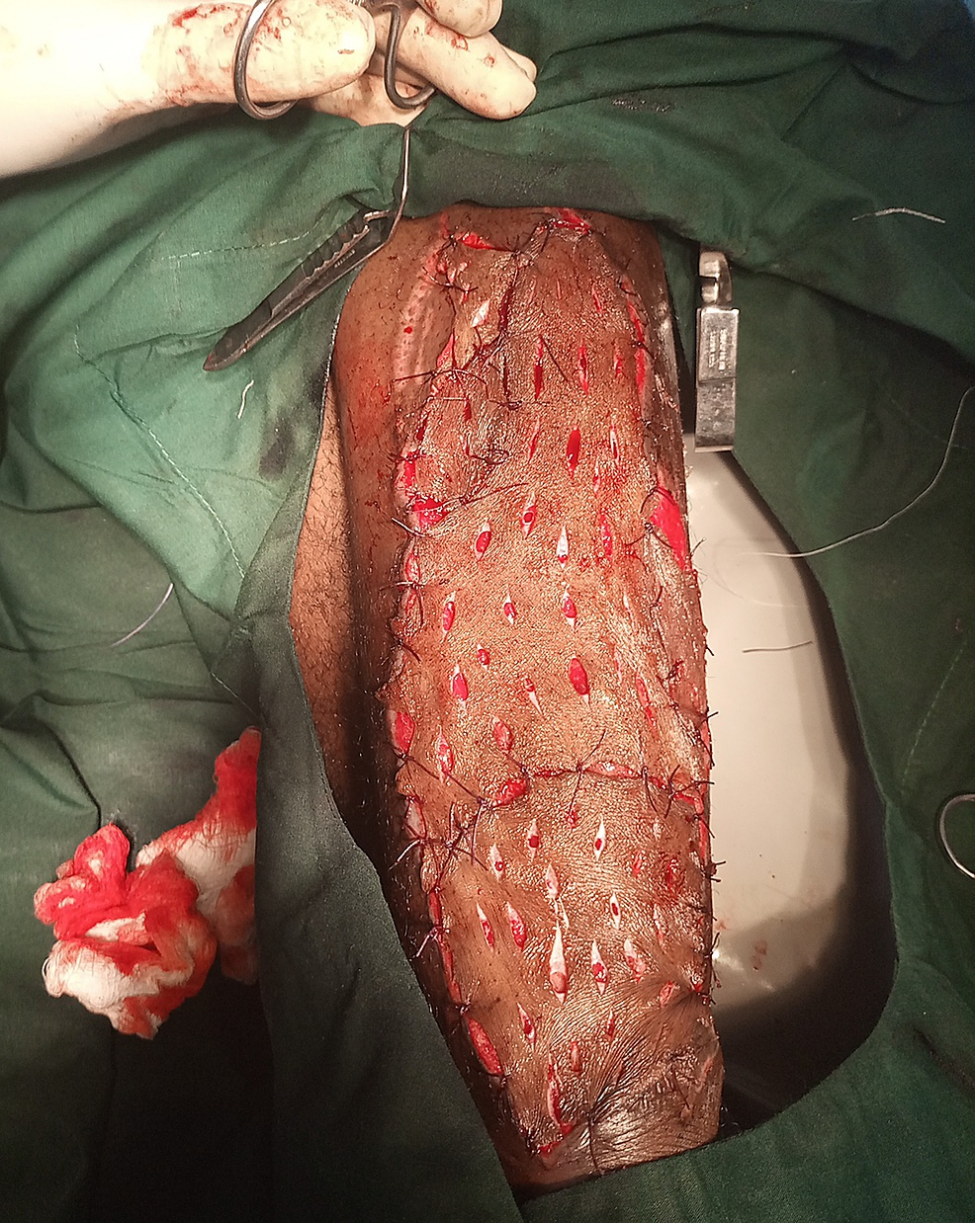
Autogenous split-thickness skin grafting of ulcer

On the sixth day of admission, the leg ulcer was irrigated with copious amounts of warm saline followed by debridement (Figure 1). Autogenous split-thickness skin grafting was performed the next day (Figure 2) while awaiting the culture results.

**Figure 3:**
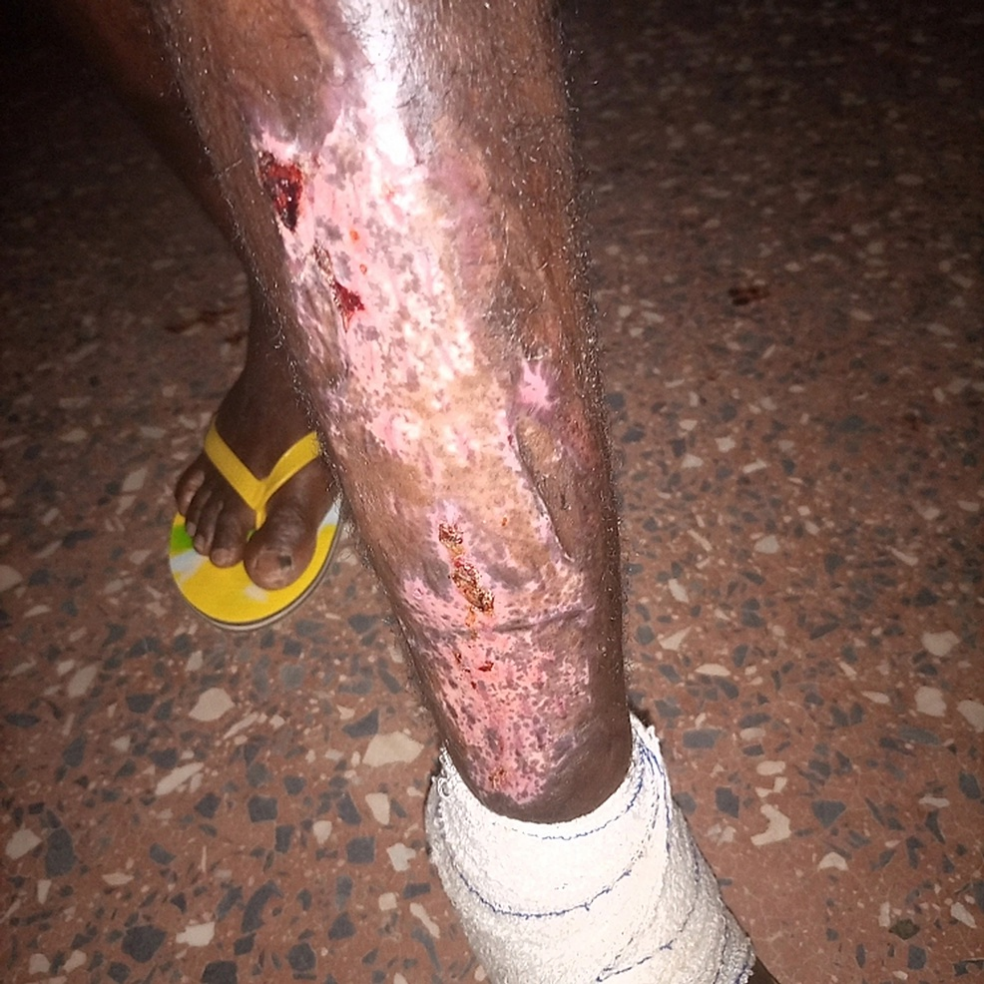
Good healing of graft site

**Figure 4:**
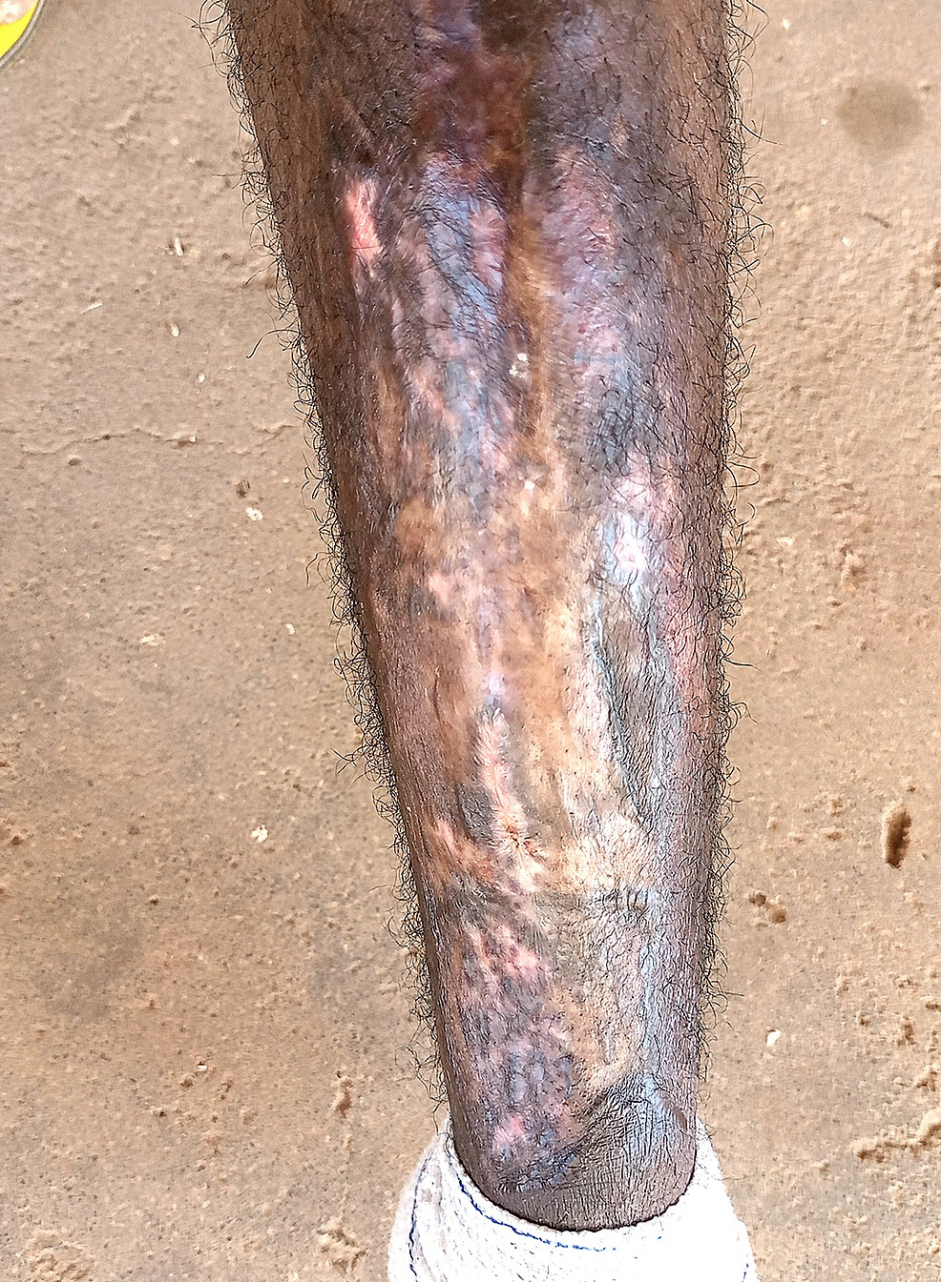
Graft site at two-week follow-up visit showing near-complete healing

On the twelfth day after initial admission, the patient was still febrile with a temperature of 38.9oC and mildly lethargic. Wound inspection revealed good graft healing with only mild serous discharge. Pre-operative blood culture results, now available, revealed B. cepacia complex sensitive only to meropenem with intermediate susceptibility but resistance to penicillin, co-trimoxazole, fluoroquinolones, cefepime, metronidazole, and ceftazidime/avibactam. Wound swab cultures showed B. cepacia with a similar sensitivity profile as the blood cultures and co-infection with Actinomyces sensitive to carbapenems but resistant to metronidazole. The patient’s diagnosis of sepsis due to an open wound was revised to B. cepacia complex bacteremia from an open wound focus. He was transferred to a private ward where he was maintained on 1 g of intravenous meropenem every eight hours, intravenous fluids, and regular inspection/dressing of the graft site. After five days of intravenous meropenem, repeat blood cultures were taken. The patient was now afebrile, breathing normally, with good healing of the graft site (Figure 3). He was discharged and followed up two weeks later. At the follow up, the patient showed complete resolution of systemic symptoms, with an excellent clinical status and near-complete healing of the graft site (Figure 4). The repeat blood cultures taken at the time of discharge were negative.

## Discussion

The Burkholderia cepacia complex is known to produce an effective outer penetration barrier with restrictive porin proteins, effective efflux pumps, and beta-lactamases to limit the potency of commonly used antibiotics. It is also associated with the evolution of altered bacterial DNA (Deoxyribonucleic acid) gyrase enzyme and dihydrofolate reductase targets. These alterations have been associated with resistance to quinolones, coumarins, cyclothialidines, pyrimethamine, and trimethoprim. The B. cepacia complex is mostly found in soil and wet environments. Burkholderia colonization is common in cystic fibrosis patients [1,2]. The B. cepacia complex is characterized by colonization rather than infection. Thus, the present case in which the complex was responsible for bacteremia is remarkable, especially in an immunocompetent adult [2,3]. Despite its well-known antibiotic resistance profile, studies have described good susceptibility of B. cepacia to ceftazidime-avibactam and trimethoprim-sulfamethoxazole and resistance to fluoroquinolones and penicillin [4]. 

Actinomycetes are a group of anaerobic bacteria predominantly found in soil, water bodies, and plant remains [5]. Actinomycetes contamination can occur due to open wound exposure to soil. They are associated with extensive local tissue damage, which facilitates deep tissue invasion and dissemination of bacteria. In the setting of co-infection, Actinomycetes spp. may contribute to cross-resistance to multiple antibiotics, thereby necessitating longer treatments or higher doses of typical antibiotics [6]. As Actinomyces spp. are not known to produce beta-lactamases, they are generally considered sensitive to beta-lactams, especially penicillin G or amoxicillin, although some species show resistance to cephalosporins [7]. The impact of polymicrobial infection, especially co-infection with other organisms with equally extensive antibiotic resistance, on the antibiotic resistance profile of these organisms remains unclear. There is a need for local tissue decontamination, a multidisciplinary approach, and to increase antibiotic treatment durations in cases of polymicrobial wound infections. The use of split-thickness skin grafting following extensive debridement of large ulcers with atypical microbial colonization has been reported to be associated with successful outcomes [8,9].

## Conclusions

There is much published research on the antibiotic resistance profiles of atypical organisms such as Burkholderia and Actinomycetes spp. and the likely mechanisms underlying resistance. However, information on the mechanisms underlying cross-resistance among these organisms is lacking. More studies on the mechanisms of cross-resistance are needed to aid the management of complex polymicrobial infections.

## References

[1] Rhodes KA, Schweizer HP (2016). Antibiotic resistance in Burkholderia spp.. Drug Resist Update.

[2] Abdelfattah R, Al-Jumaah S, Al-Qahtani A, Al-Thawadi S, Barron I, Al-Mofada S (2018). Outbreak of Burkholderia cepacia bacteraemia in a tertiary care centre due to contaminated ultrasound probe gel. J Hosp Infect.

[3] Mahenthiralingam E, Campbell ME, Henry DA, Speert DP (1996). Epidemiology of Burkholderia cepacia infection in patients with cystic fibrosis: analysis by randomly amplified polymorphic DNA fingerprinting. J Clin Microbiol.

[4] Van Dalem A, Herpol M, Echahidi F (2018). In Vitro Susceptibility of Burkholderia cepacia complex Isolated from Cystic Fibrosis Patients to Ceftazidime-Avibactam and Ceftolozane-Tazobactam. Antimicrob Agents Chemother.

[5] Bhatti AA, Haq S, Bhat RA (2017). Actinomycetes benefaction role in soil and plant health. Microb Pathog.

[6] Smith AJ, Fhogartaigh CN, Millar M (2016). Is the presence of Actinomyces spp. in blood culture always significant?. J Clin Microbiol.

[7] Valour F, Sénéchal A, Dupieux C (2014). Actinomycosis: etiology, clinical features, diagnosis, treatment, and management. Infect Drug Resist.

[8] AlShehri YA, AlBurshaid H, AlBassam L, AlMutairi K (2019). Management of Fournier's gangrene with skin grafting by bagging technique of testes: case report. GMS Interdiscip Plast Reconstr Surg DGPW.

[9] Koschel S, Manning TG, Perera M (2017). Successful split thickness skin grafting in the presence of heavy colonisation with rare bacterium Aeromonas hydrophila: a case report. JPRAS Open.

